# Supplementary material for: Modeling glioblastoma heterogeneity as a dynamic network of cell states
Source: Mol Syst Biol. 2021 Sep 16;17(9):e10105. doi: 10.15252/msb.202010105 (PMC8444284; doi:10.15252/msb.202010105)
Supplement: Supplementary file 5 — Source Data for Figure 3 [file MSB-17-e10105-s001.zip › Figure3A_sourcedata/GSEA_3065/hallmarks_state6.GseaPreranked.1623416308702/HALLMARK_CHOLESTEROL_HOMEOSTASIS.html]

Details for gene set HALLMARK\_CHOLESTEROL\_HOMEOSTASIS[GSEA]

|  || Dataset | state6 |
| Phenotype | NoPhenotypeAvailable |
| Upregulated in class | na\_neg |
| GeneSet | HALLMARK\_CHOLESTEROL\_HOMEOSTASIS |
| Enrichment Score (ES) | -0.52646536 |
| Normalized Enrichment Score (NES) | -1.9018414 |
| Nominal p-value | 0.0 |
| FDR q-value | 8.4525463E-4 |
| FWER p-Value | 0.003 |
Table: GSEA Results Summary

  

Fig 1: Enrichment plot: HALLMARK\_CHOLESTEROL\_HOMEOSTASIS      
 Profile of the Running ES Score & Positions of GeneSet Members on the Rank Ordered List

  

| PROBE | GENE SYMBOL | GENE\_TITLE | RANK IN GENE LIST | RANK METRIC SCORE | RUNNING ES | CORE ENRICHMENT || 1 | PNRC1 |  |  | 123 | 0.202 | 0.0259 | No |
| 2 | TP53INP1 |  |  | 342 | 0.145 | 0.0296 | No |
| 3 | ATF3 |  |  | 494 | 0.124 | 0.0368 | No |
| 4 | ERRFI1 |  |  | 1210 | 0.075 | -0.0302 | No |
| 5 | ACSS2 |  |  | 1453 | 0.066 | -0.0449 | No |
| 6 | CTNNB1 |  |  | 1461 | 0.066 | -0.0327 | No |
| 7 | STX5 |  |  | 1508 | 0.064 | -0.0254 | No |
| 8 | PMVK |  |  | 1723 | 0.056 | -0.0389 | No |
| 9 | ANXA5 |  |  | 1978 | 0.048 | -0.0584 | No |
| 10 | SREBF2 |  |  | 2018 | 0.047 | -0.0535 | No |
| 11 | JAG1 |  |  | 2234 | 0.040 | -0.0702 | No |
| 12 | S100A11 |  |  | 2523 | 0.033 | -0.0967 | No |
| 13 | ANTXR2 |  |  | 2981 | 0.022 | -0.1445 | No |
| 14 | ECH1 |  |  | 3187 | 0.018 | -0.1644 | No |
| 15 | GNAI1 |  |  | 3240 | 0.017 | -0.1671 | No |
| 16 | LSS |  |  | 3264 | 0.016 | -0.1665 | No |
| 17 | PLAUR |  |  | 3956 | 0.001 | -0.2453 | No |
| 18 | CXCL16 |  |  | 3974 | 0.001 | -0.2470 | No |
| 19 | FDFT1 |  |  | 3979 | 0.001 | -0.2473 | No |
| 20 | FASN |  |  | 4033 | 0.000 | -0.2533 | No |
| 21 | PCYT2 |  |  | 4540 | -0.010 | -0.3093 | No |
| 22 | ETHE1 |  |  | 4610 | -0.011 | -0.3151 | No |
| 23 | LGMN |  |  | 4983 | -0.018 | -0.3541 | No |
| 24 | HMGCS1 |  |  | 5446 | -0.027 | -0.4016 | No |
| 25 | HSD17B7 |  |  | 5531 | -0.029 | -0.4055 | No |
| 26 | MVK |  |  | 5540 | -0.029 | -0.4006 | No |
| 27 | ABCA2 |  |  | 5665 | -0.032 | -0.4085 | No |
| 28 | CPEB2 |  |  | 5862 | -0.035 | -0.4239 | No |
| 29 | MVD |  |  | 6012 | -0.038 | -0.4334 | No |
| 30 | TM7SF2 |  |  | 6044 | -0.039 | -0.4293 | No |
| 31 | PLSCR1 |  |  | 6134 | -0.041 | -0.4313 | No |
| 32 | SC5D |  |  | 6168 | -0.042 | -0.4268 | No |
| 33 | NSDHL |  |  | 6236 | -0.043 | -0.4259 | No |
| 34 | FADS2 |  |  | 6359 | -0.046 | -0.4308 | No |
| 35 | CHKA |  |  | 6526 | -0.050 | -0.4398 | No |
| 36 | ATF5 |  |  | 6981 | -0.064 | -0.4791 | No |
| 37 | GLDC |  |  | 7032 | -0.066 | -0.4718 | No |
| 38 | CYP51A1 |  |  | 7511 | -0.082 | -0.5103 | Yes |
| 39 | EBP |  |  | 7564 | -0.084 | -0.4996 | Yes |
| 40 | LGALS3 |  |  | 7566 | -0.084 | -0.4830 | Yes |
| 41 | FDPS |  |  | 7587 | -0.085 | -0.4684 | Yes |
| 42 | LDLR |  |  | 7648 | -0.088 | -0.4579 | Yes |
| 43 | TNFRSF12A |  |  | 7784 | -0.094 | -0.4547 | Yes |
| 44 | DHCR7 |  |  | 7806 | -0.096 | -0.4381 | Yes |
| 45 | IDI1 |  |  | 7851 | -0.099 | -0.4236 | Yes |
| 46 | ACAT2 |  |  | 7868 | -0.099 | -0.4058 | Yes |
| 47 | SCD |  |  | 7908 | -0.102 | -0.3902 | Yes |
| 48 | TMEM97 |  |  | 8049 | -0.110 | -0.3845 | Yes |
| 49 | ALCAM |  |  | 8323 | -0.135 | -0.3891 | Yes |
| 50 | SEMA3B |  |  | 8382 | -0.144 | -0.3672 | Yes |
| 51 | CLU |  |  | 8387 | -0.145 | -0.3391 | Yes |
| 52 | FABP5 |  |  | 8414 | -0.148 | -0.3128 | Yes |
| 53 | ATXN2 |  |  | 8533 | -0.169 | -0.2929 | Yes |
| 54 | GUSB |  |  | 8561 | -0.176 | -0.2613 | Yes |
| 55 | LPL |  |  | 8638 | -0.208 | -0.2289 | Yes |
| 56 | HMGCR |  |  | 8759 | -0.301 | -0.1832 | Yes |
| 57 | CD9 |  |  | 8762 | -0.303 | -0.1236 | Yes |
| 58 | SQLE |  |  | 8767 | -0.319 | -0.0609 | Yes |
| 59 | GPX8 |  |  | 8771 | -0.324 | 0.0029 | Yes |
Table: GSEA details [plain text format]

  

Fig 2: HALLMARK\_CHOLESTEROL\_HOMEOSTASIS: Random ES distribution      
 Gene set null distribution of ES for **HALLMARK\_CHOLESTEROL\_HOMEOSTASIS**
